# Influences of genetically predicted and attained education on geographic mobility and their association with mortality

**DOI:** 10.1016/j.socscimed.2023.115882

**Published:** 2023-03-31

**Authors:** Elsa Ojalehto, Deborah Finkel, Tom C. Russ, Ida K. Karlsson, Malin Ericsson

**Affiliations:** a Department of Medical Epidemiology and Biostatistics, Karolinska Institutet, Stockholm, Sweden; b Center for Economic and Social Research, University of Southern California, Los Angeles, CA, USA; c Aging Research Network – Jönköping, School of Health and Welfare, Jönköping University, Jönköping, Sweden; d Alzheimer Scotland Dementia Research Centre, University of Edinburgh, Edinburgh, UK; e Division of Psychiatry, Centre for Clinical Brain Sciences, University of Edinburgh, Edinburgh, UK; f Aging Research Center, Karolinska Institutet & Stockholm University, Stockholm, Sweden

**Keywords:** Polygenic score, Attained education, Geographic mobility, Mortality, Socioeconomic status

## Abstract

**Introduction:**

Both educational attainment and genetic propensity to education (PGS_Edu_) have been associated with geographic mobility. Socioeconomic conditions are, in turn, associated with individuals’ health. Geographic mobility could therefore lead to better health for some since it could provide better opportunities, like education. Our aim was to study how attained education and genetic predisposition for higher education are related to geographic mobility, and how they affect the association between geographic mobility and mortality.

**Methods:**

We used data from the Swedish Twin Registry (twins born 1926–1955; n = 14,211) in logistic regression models to test if attained education and PGS_Edu_ predicted geographic mobility. Cox regression models were then performed to test if geographic mobility, attained education, and PGS_Edu_ were associated with mortality.

**Results:**

The results show that both attained education and PGS_Edu_ predicted geographic mobility, in both independent and joint effect models, with higher education associated with higher mobility. Geographic mobility was associated with lower mortality in the independent effect model, but joint effect models showed that this association was completely explained by attained education.

**Conclusions:**

To conclude, both attained education and PGS_Edu_ were associated with geographic mobility. Moreover, attained education explained the relationship between geographic mobility and mortality.

## 1. Introduction

Socioeconomic status (SES) plays a significant role in health differences across the world, indicating that adults with lower SES have a higher risk of falling ill or dying prematurely (Mackenbach et al., 2008). Studies also indicate associations between the health of individuals and conditions in their area of residence meaning that geographic mobility might play a role in health differences, as people who live in more deprived areas tend to have poorer health (Curtis et al., 2009). Geographic mobility could therefore lead to better living conditions, including opportunities in both education and work, raising the question about how geography and SES affect health.

## 2. Background

### 2.1. Educational influences on late-life health inequalities

The aging population has increased in recent decades and a greater proportion survives to the oldest ages, a trend that is predicted to continue (Organization, 2021). This demographic change is due to both increased life expectancy and improved survival in younger ages, predominantly because of the worldwide historic socioeconomic development during the past 50 years (Organization, 2021).

Education is often used as a socioeconomic indicator for studying health gradients (Smith, 2007). Adverse health outcomes and shorter lifespans are more common in lower socioeconomic groups whether these are defined by education, income, or occupational class (Ericsson, 2019; Huisman et al., 2004). For example, many longitudinal studies have shown that adults with lower education have a higher risk of falling ill or dying prematurely (Mackenbach et al., 2008). Research on human health has stated that education promotes both non-cognitive and cognitive skill formation that could contribute to engagement in a lifestyle that includes health promoting activities (Hoffmann et al., 2019). Furthermore, education has other beneficial health consequences. In particular, it can provide access to full-time, fulfilling work and secures an adequate income that helps prevent economic hardship (Mirowsky et al., 2005).

In all countries, mortality rates are higher among those who live in less advantaged socioeconomic areas (Mackenbach et al., 2015). According to Link and Phelan’s (Phelan et al., 2004) theory of SES as a “fundamental cause” of mortality inequalities, SES is most directly associated with ‘preventable’ mortality. Specifically, one study found a faster decline in mortality over time among highly educated groups in preventable causes of death than non-preventable causes of death (Mackenbach et al., 2017). This decline remained after adjusting for familial confounding in twin-studies (Ericsson et al., 2019).

### 2.2. Socioeconomic and sex differences in educational opportunities and health

Historically, opportunities to access education and waged work have been severely limited for women and those in lower socioeconomic groups in Sweden. An educational expansion during the 1950s and 1960s meant that higher education became more available for all instead of being reserved for the upper social classes (Larsson and Westberg, 2015). These educational reforms shifted the old, privileged society to a welfare state. This shift gradually increased the educational opportunities for women and those in lower social classes as it led to large-scale countrywide reformation between 1949 and 1962 where the compulsory school reform was implemented and extended the basic education gradually for every municipality to provide equal opportunities for all children (Okbay, 2017).

### 2.3. Genetic influences on educational attainment

While variation in educational achievement is partly influenced by parental socioeconomic position, educational achievement is also strongly dependent on the individual’s own cognitive ability during childhood and adolescence. Even though children’s cognitive abilities are partly dependent on their rearing environment, genetic influences are also highly influential (Plomin and Deary, 2015). Thus, genetic influences are also highly influential on educational attainment (Deary et al., 2007; Plomin and Deary, 2015).

Empirical evidence derived from genome-wide association studies (GWAS), examining genetic associations across the genome in association to a trait, have identified thousands of genetic variants related to education (Lee et al., 2018; Okbay et al., 2022). Polygenic scores (PGS) are a weighted combination of these individual genetic variants – each with a very small effect. Rather than being seen as a gene for education, PGS_Edu_ can be explained as a normally distributed genetic continuum. Some individuals carry few genetic variants that are associated with higher educational achievement, most of the population carries some such variants, and a few carry many variants. PGS_Edu_ has been shown to predict educational attainment in cohorts in three continents and has even explained differences in educational attainment between siblings from the same family (Belsky et al., 2016). PGS_Edu_ explains 15% of the inter-individual differences in educational achievement (Okbay et al., 2022). The GWAS used in this study measured a joint analysis of educational attainment and three related cognitive phenotypes that generated a polygenic score that explain 11–13% of the variance in educational attainment (Lee et al., 2018).

Genetically-influenced differences in educational achievement have increased over time, possibly because of the declining influence of environmental barriers, like the educational reforms implemented in Sweden during the 1950s. This increase in genetically-influenced differences is shown in twin studies which report greater heritability of educational achievement among twins born more recently, when the introduction of equality in educational policies have reduced variation due to shared-environmental factors (Colodro-Conde et al., 2015). Between-country comparisons show that the heritability of educational achievement is greater in countries where the environment puts less limitations on educational achievement, as in the Nordic countries (Branigan et al., 2013).

## 3. Education, genetics, and geographic mobility

Previous studies indicate that there are both theoretical and observed associations between the health of individuals and the conditions of the area they live in. Specifically, individuals living in more deprived areas tend to have poorer health (Curtis et al., 2009). Research shows that both educational attainment and genetic propensity to education can be associated with geographic mobility (Abdellaoui et al., 2019; Belsky et al., 2016, 2019; Breen, 2004; Chen and Rosenthal, 2008). There are many reasons why individuals choose to move location. Workers move to cities with improving occupational environments, especially people with higher levels of human capital (e.g. education) (Chen and Rosenthal, 2008). In the Dunedin study, participants with high PGS_Edu_ were geographically mobile in search for occupational opportunities, built more successful careers, found life-partners with higher social status, and built stronger foundations for retirement (Belsky et al., 2016).

### 3.1. Life-course epidemiology, social causation, and indirect selection

When interpreting the interplay between education and health, a life-course perspective is required to understand socioeconomic influences on health and survival. The *social causation hypothesis* assumes that there is a direct effect of socioeconomic influence on health, where SES influences health through different behaviors and pathways directly caused by SES (Ericsson, 2019; Hoffmann et al., 2019). In contrast, the *indirect selection hypothesis* assumes that there is no causal relationship between SES and health, but rather that some underlying factor is accountable for their association (Foverskov and Holm, 2016). Underlying confounders would therefore be plausible determinants for both SES and health such as genetic factors like intelligence and personality (Foverskov and Holm, 2016).

The current study aimed to increase the understanding of how education influences geographic mobility, and if mobility, in turn, influences the risk of mortality. By using rich life-course data from the Swedish Twin Registry, we investigated the effect of both genetically predicted and attained education on geographic mobility, and how education and geographic mobility together predict health outcomes (all-cause mortality). Due to the extensive reformation of the educational system occurring in Sweden in the 1950’s, different cohorts have had different access to education, especially among women and those with lower SES (Larsson and Westberg, 2015; Okbay, 2017). As genetic variation, and thereby the interpretation of a PGS for education which is constant across generations, considering genetic predisposition to educational attainment increases the possibility to measure the effects on health and to disentangle direct and indirect effects.

We investigated three research questions: 1) Does educational attainment influence geographic mobility? 2) Does genetic predisposition to higher education influence geographic mobility? 3) Does geographic mobility decrease the risk of mortality, and is that association explained by educational factors? In addition, we investigated if there are sex and cohort differences, to further understand how historical differences may impact the associations.

## 4. Methods

### 4.1. Data

This retrospective longitudinal cohort study used data from the Swedish Twin Registry (STR), with twins born in the middle cohort (P. Lichtenstein et al., 2002; Paul Lichtenstein et al., 2006; Zagai et al., 2019). The middle cohort was compiled in 1970, with the help of national birth registers. All same-sex pairs born 1926–1958 were contacted through a questionnaire in 1973 (Q73), when about 36,000 individuals responded (P. Lichtenstein et al., 2002). The questionnaire contained questions regarding twin and family information, demographic information (education and occupation), environmental exposures, and other questions concerning health, lifestyle, and psychosocial traits. In addition, the questionnaire included a question on all places the twin had lived since childhood until that time-point (Ericsson, 2019; P. Lichtenstein et al., 2002).

After Q73, several additional data collections have occurred within the STR. The largest was the Screening Across the Life Span study (SALT), an extensive computer assisted telephone interview that was carried out 1998 to 2002 (P. Lichtenstein et al., 2002; Paul Lichtenstein et al., 2006). It was aimed at all Swedish twins (same-sex and opposite-sex pairs) born 1958 or earlier. Out of the total 52,080 twins from the old and the middle cohort that were contacted, a total of 44,919 responded (P. Lichtenstein et al., 2002; Paul Lichtenstein et al., 2006; Zagai et al., 2019). The response rate was 74% for the middle cohort (Pedersen et al., 2002). Subsets of the SALT sample participated in the TwinGene and SALT-Y studies, conducted 2004–2008 and 2009–2010, respectively, which included collection of blood samples or saliva for genotyping (Zagai et al., 2019). Of particular value, the STR is linked to other national registers in Sweden (Ericsson, 2019).

The current study population is based on individuals who participated in both Q73 and SALT. We excluded individuals from the middle cohort born 1956–1958, as they were under the age of 18 in 1973 (n = 1633) and might not have had the chance to leave home yet, and 29 individuals were excluded due to missing data. The final study population consisted of 14,211 individuals born 1926–1955. In this sample, educational attainment data was available for 13,858 individuals and genetic data (PGS_Edu_) for 8094 individuals. Data on both educational attainment and PGS_Edu_ were available for 7741 individuals.

### 4.2. Variables

*Educational attainment* was retrieved from SALT and measured by levels of education, divided to five different categories: “primary”, “lower secondary”, “upper secondary/post-secondary non tertiary”, “short-cycle tertiary”, and “university degree (bachelor’s or above)” (Eurostat, 2015).

*PGS*_*Edu*_ was computed using data collected as part of TwinGene and SALT-Y (Zagai et al., 2019), based on the GWAS from Lee and colleagues, a study on 1.1 million individuals (Lee et al., 2018). Polygenic scores are created by summing the number of associated alleles (0, 1 or 2) at each position in the genome, weighted by their effect in a GWAS of the trait. The resulting score is a linear measure of genetic propensity to a trait, e.g. educational attainment. Prior to analyses, the PGS in this dataset was standardised with mean = 0 and SD = 1 to increase interpretability, as regression coefficients then represent the effect of one SD higher PGS. The generation of the PGS_Edu_ is described in detail in the Supplementary section.

*Geographic mobility,* which is defined as the geographical movement of people (Haley, 2017), was obtained from the Q73 questionnaire. The questionnaire included questions on every place the twin had lived from birth through 1973, according to county, municipality, and parish [“län, kommun och församling_”_ in Swedish]. We defined geographic mobility as moving across county lines (1 = mobility), compared to not having moved across county lines (0 = no mobility), at any point from age 15 to 1973. There are 21 counties in Sweden today. However, during the study period there were 25 counties in Sweden and that is the definition we used.

*All-cause mortality* information was available through linkage to the Population Register from the Swedish Tax Agency. These data are updated monthly and include the date of death for all Swedish residents (Befolkningsstatistik, 2022).

#### Other covariates:

Additional covariates were considered as appropriate. *Age and sex*: Everyone born 1926–1955 was included in the analyses, and age in years was included in all analyses. The analyses also included the individual’s sex at birth retrieved from the registers (1 = men, 2 = women). *Cohort*: To investigate cohort differences in the total sample we divided them to into subsamples: born between 1926 and 1940 (cohort 1, N = 4873) and born between 1941 and 1955 (cohort 2, N = 9338). *Principal components:* The genetic backgrounds of individuals differ across as well as within countries, and this population stratification can introduce bias to models of e.g. PGS (Pärna et al., 2022). To account for such bias, we computed principal components based on the individual genetic data and included the first five as covariates in all models including the PGS_Edu_ to control for population stratification. *Parental SES*: To incorporate other family characteristics than the PGS_Edu_, we conducted sensitivity analyses that were adjusted for parental SES. Rearing social class data were harmonized as a three-level classification (Social group I, II, III), in SALT, parental occupation was retrieved from birth journals (Magnusson et al., 2013).

### 4.3. Statistical analysis

First, we investigated how well PGS_Edu_ predicted attained education, using a linear regression model. Second, we measured the differences of the effect of PGS_Edu_ on attained education stratified by mobility status, by including an interaction term between PGS_Edu_ and mobility. To investigate the influence of attained education and PGS_Edu_ on geographic mobility, logistic regression analyses were performed, with attained education and PGS_Edu_ as predictors of geographic mobility (0 = not mobile, 1 = mobile), in both independent and joint effect models.

Furthermore, we investigated if geographic mobility influenced all-cause mortality (here used as a general measure of health) and if this relationship was confounded by attained education or PGS_Edu_. We used Cox proportional hazard regression to compare mortality between those who were and were not geographically mobile, and to test if the association was influenced by educational level or PGS_Edu_. We used age (in years) as the underlying time scale, following individuals from when they entered the study (age at 1973) and until either the individual’s death year (event) or end of the follow-up (December 2021).

In sensitivity analyses, we tested if the observed associations were similar for those who moved after age 18 instead of after age 15 and who were aged at least 25 in 1973, to exclude those who moved for their education. To examine any differences between sex and cohorts, the analyses were repeated stratifying the sample separately by sex and birth cohort. To further examine family characteristics other than the PGS_Edu,_ we adjusted the main models for parental SES in sensitivity analyses. Since the sample size differed between both PGS_Edu_ and attained education, we did sensitivity analyses on the main models with this restrictive sample for those with information on both variables (PGS_Edu_ and attained education).

All models were adjusted for sex and birth year. All models including the PGS_Edu_ were also adjusted for population stratification.

The statistical software STATA (v15.1) (StataCorp, 2015) was used for all statistical analyses.

### 4.4. Ethical considerations

Data in STR are pseudonymized which means that only data with serial numbers are analyzed and available, without identifying information such as names and addresses. To minimize the risk of the possibility of identification through backtracking, this project ensured that only limited data were available, such that the researchers had access only to the data needed for analyses. Additionally, informed consent was obtained when data were collected and the study was approved by the Regional Ethics Board at Karolinska Institutet, Stockholm.

## 5. Results

The descriptive statistics are presented in Table 1. A majority of the study population was not geographically mobile (had not moved counties between birth and 1973). Geographically mobile men had the largest proportion of university education. Geographically mobile women were more likely to have had lower secondary school or university education. PGS_Edu_ was higher for those who were geographically mobile than those who were not, for both men and women. Later birth cohorts were more geographically mobile than earlier cohorts.

### 5.1. Association between PGS_Edu_ and attained education

The linear regression model measured the association between the PGS_Edu_ and attained education. The association was stronger in those who were geographically mobile (β = 0.49, 95% CI 0.38–0.48) than in those who were not mobile (β = 0.25, 95% CI 0.22–0.29). The sex and cohort stratified results showed similar results, and are presented in Supplementary Table S1.

### 5.2. Geographic mobility as a function of attained education and PGS_Edu_

The logistic regression model, presented in Table 2, investigated the association between attained education and PGS_Edu_ in relation to geographic mobility. The independent effect model includes either attained education or PGS_Edu_, and the joint effect model both attained education and PGS_Edu_, thus adjusting for each other.

In the unadjusted independent effect model, every unit increase in attained education was associated with a 32% higher chance of geographic mobility. This association was stronger after adjusting for sex and birth year. Every SD increase in PGS_Edu_ was associated with 28% higher chance of geographic mobility, and the results were stable after adjusting for sex and birth year. In the joint effect model, the effect of attained education was unaffected, while that of the PGS_Edu_ was attenuated to 12%.

Table 2 also presents the results divided by cohorts and sex. In all subsamples, the association between attained education and geographic mobility remained stable in the joint effect models, while the influence of PGS_Edu_ was attenuated, similar to the main model. In the unadjusted independent effect model of the 1926–1940 cohort, every unit increase in attained education was associated with 74% higher geographic mobility for men, and 62% higher for women with no difference in the adjusted model. In the 1940–1955 cohort, every unit increase in attained education was associated with 53% (men) and 26% (women) higher chance of geographic mobility in the unadjusted independent effect model, with higher estimates in the adjusted model.

### 5.3. Risk of mortality as a function of geographic mobility, attained education and PGS_Edu_

**Fig. 1.**
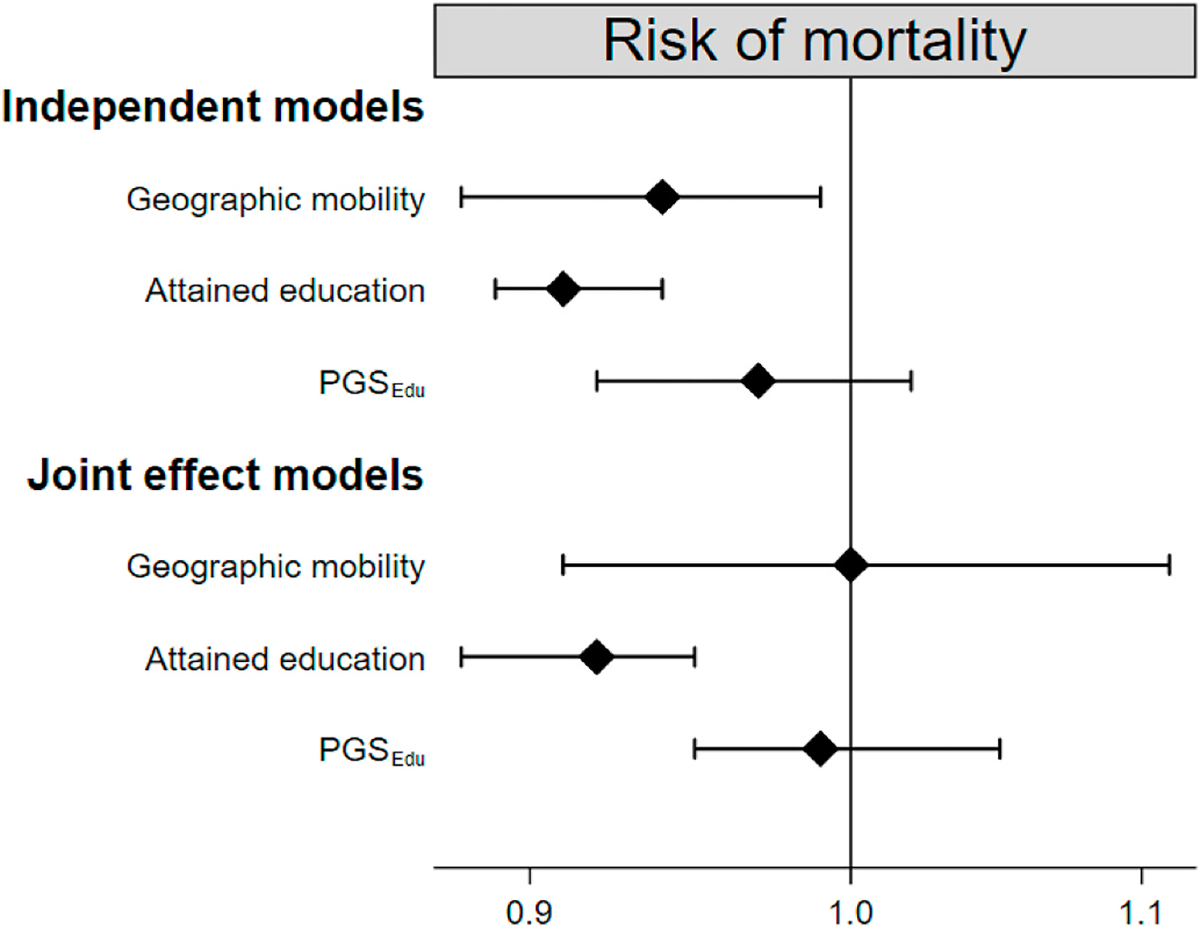
Cox regression investigating the risk (hazard rate ratios and 95% confidence intervals) of mortality as a function of geographic mobility, attained education and PGS_Edu_. All models are adjusted for sex and birth year. *PGS*_*Edu*_ polygenic score for education.

The Cox regression model, investigated geographic mobility and educational factors (genetic and attained) in relation to mortality risk. There were no indications of violation of the proportional hazard assumption. The independent effect models contains either geographic mobility, attained education or PGS_Edu_. The joint effect models contain geographic mobility, attained education and PGS_Edu_ together in the same model, thus mutually adjusted for. Fig. 1 presents the results for both independent and joint effect model. In the independent effect model, geographic mobility was associated with 6% lower mortality (HR = 0.94, 95% CI 0.88–0.99) and attained education with 9% lower risk of mortality (HR = 0.91, 95% CI 0.89–0.94), while the PGS_Edu_ had no influence on mortality risk (HR = 0.97, 95% CI 0.92–1.02). In joint effect models, geographic mobility was no longer associated with the risk of mortality (HR =1.00, 95% CI 0.91–1.11). Attained education was the only variable still associated with decreased risk of mortality (HR = 0.92, 95% CI 0.88–0.95).

**Fig. 2.**
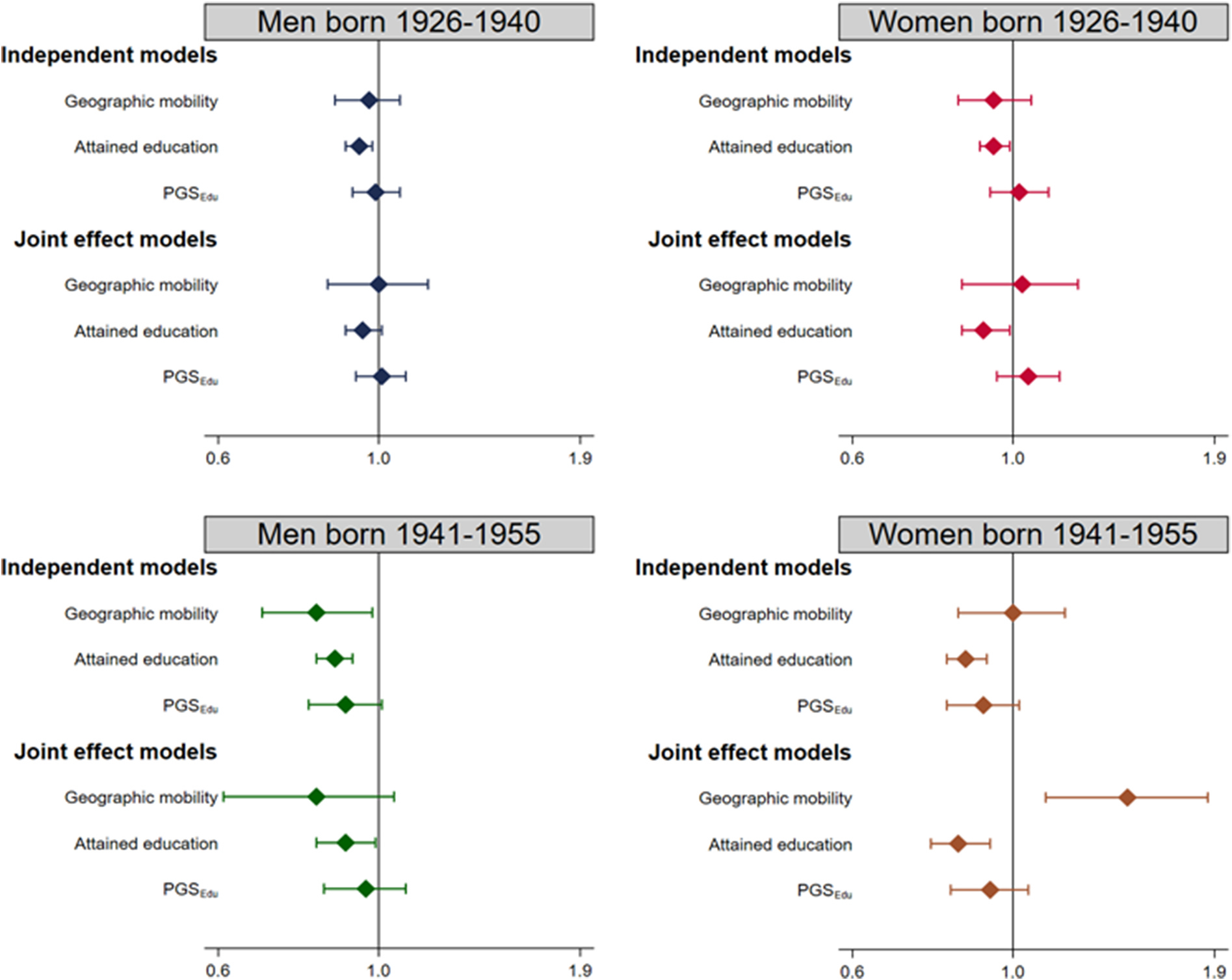
Cox regression investigating the risk (HR, 95% CI) of mortality as a function of geographic mobility, attained education and PGS_Edu_ by birth cohort and sex. All models are adjusted for birth year. Detailed estimates are presented in Supplementary table S2. *PGS*_*Edu*_ polygenic score for education.

Fig. 2 presents the results divided into cohorts and sex. The trends are similar across subgroups, and attained education is the only variable associated with lower risk of mortality in all subsamples (effect estimates are presented in Supplementary Table S2). Geographic mobility in women born in the later cohort was associated with increased risk of mortality, however follow-up analyses (presented in Supplementary Table S3) indicate that the association between geographic mobility and higher risk of mortality was only present among women with lower education.

### 5.4. Sensitivity analyses

Results from proportional hazard regression on those who moved after the age of 25 were consistent with those from the main analyses. The estimates are presented in Supplementary Table S4 for the total sample, and in Supplementary Table S5 stratified by cohort and sex. The results from sensitivity analyses incorporating parental SES (only available for a subsample) are consistent with those from the main analyses, these estimates are presented in Supplementary Tables S6 and S7. The sensitivity analyses on the main models for the restrictive sample (those with information on both variables) are presented in Supplementary Tables S8 and S9, due to the smaller sample size, precision was attenuated.

## 6. Discussion

The aim of this study was to explore educational factors, geographic mobility, and the risk of mortality, and in addition to study differences in associations across sex and cohorts. The main finding was that while geographic mobility was associated with a lower risk of mortality, this association was completely explained by educational attainment, indicating a direct effect of education.

More specifically, we investigated this relationship through three research questions. The first research question investigated the association between educational attainment and geographic mobility. We found that higher attained education was associated with higher odds of being geographically mobile. The amplified association after correcting for sex and birth year can be understood from a life-course perspective as a result of the educational reforms instituted during the 1950’s, which would have impacted later born cohorts (Breen, 2004). Indeed, stratifying by birth cohort showed an association between higher attained education and increase in geographic mobility for all subgroups when adjusting for sex and birth year, but especially for the later born cohort. In line with this, previous findings have also shown that individuals born in the middle of the twentieth century tend to be more upwardly socioeconomically mobile, including geographically mobile, compared to earlier born birth cohorts (Breen, 2004). The study population in this paper was born 1926–1955. Thus only a few of those born in the earlier cohorts had the opportunity to access higher education whereas numerous individuals born in the later cohorts likely had that opportunity (Larsson and Westberg, 2015; Okbay, 2017), something that was also seen in our results. The association between attained education and geographic mobility increased much more for the later cohort compared to the earlier cohort when it was adjusted for birth year, a result that could be attributed to the educational reforms implemented in the 1950’s. Interestingly, the association between educational level and geographic mobility was not influenced by the PGS_Edu_, indicating that attained education is associated with geographic mobility regardless of genetically predisposed education.

Second, we investigated the association between genetic propensity to education and geographic mobility. Genetic propensity to higher education was associated with higher odds of being geographically mobile. This association was not as strong as that for educational attainment, with little change after adjusting for sex and birth year. Genetics of the population are largely stable over time, and not affected by e.g. educational reforms, which may be why adjusting for sex and birth year did not affect the association. Although, the effect of genetic propensity for education on geographic mobility was attenuated in the joint effect model, it was still statistically significant, indicating that one’s genetically predicted education has an effect above and beyond that of attained education. This finding supports previous studies that individuals with high genetic propensity for education are more geographically mobile in search for educational and occupational opportunities (Belsky et al., 2016).

Third, we investigated whether geographic mobility was associated with lower risk of all-cause mortality, and if the relationship was confounded by either genetic propensity or attained educational level. Interestingly, geographic mobility was associated with lower risk of mortality in independent effect models. However, this association disappeared in the joint effect models, indicating that it is explained entirely by education. In fact, only educational attainment was associated with lower risk of mortality in both independent and joint effect models, indicating a direct effect of education on mortality. As mentioned in the introduction, the social causation and indirect selection could serve as theoretical explanations of our results. This result is in line with the social causation hypothesis which posits a direct effect of socioeconomic influence on health (Hoffmann et al., 2019). Genetic propensity for education was not statistically significant in either the independent or joint effect model. Therefore, no support was found for the indirect selection hypothesis that PGS_Edu_ served as an underlying factor accountable for the association between SES and health (Foverskov and Holm, 2016). This finding is important as it reassuringly indicates that genetic predisposition is not deterministic of ones attained educational level.

This study provides further evidence that social pathways such as geographic mobility decrease the risk of adult mortality, but that the relationship is explained by attained educational level. Thus, the geographical move itself does not explain the association, but it may be explained by what the move could entail in terms of other opportunities, brought about by a higher education. Our results agree with previous findings and provide support for theories that socioeconomic status is a fundamental cause of health inequalities (Phelan et al., 2004). A person’s socioeconomic status, influenced by their educational level, provides them with flexible resources to avoid disease risks and the consequences of those risks, regardless of the circumstances (Mackenbach et al., 2015; Phelan et al., 2004).

Surprisingly, geographically mobile women in the later born cohort had an increased risk of mortality, but follow up analyses showed that it was only among those with lower education, with the association decreasing for every educational level. This result raises the question of why these sex differences are seen. Interestingly, previous research has stated that educational attainment coincides with occupation and chances of occupational gains, especially for women (Härkönen and Bihagen, 2011). In addition, life expectancy for women with lower education has not increased at the same rate as for women with higher education (Fors et al., 2021; “Socialstyrelsen, 2020 Vård och omsorg om äldre,” 2020). It is possible that geographically mobile women followed their husbands, since higher educational and occupational opportunities were more available to men (Larsson and Westberg, 2015; Okbay, 2017), especially for the earlier born cohorts. It ultimately raises the question of whether the job opportunities for women with low education actually increased their risk of mortality. They might not have had the opportunity for occupational gains, therefore worked in blue-collar occupations, which could be one of many explanations why geographically mobile women with low educational attainment in the later cohort had an increased risk of mortality.

### 6.1. Strengths and limitations

The main strength of this paper is the large study population with long follow up. We used a rich dataset from the STR with a large variety of variables such as, genetic propensity to education, attained education, geographic mobility, and mortality. These data provided a unique opportunity to disentangle social, genetic, and environmental factors, going beyond simple associations to multivariable analyses (Mishra et al., 2015). By using longitudinal data, we could examine long-term effects of educational attainment and geographic mobility (Mishra et al., 2015). Another strength is the use of mortality as the health outcome measurement since it is a robust measure of health, and through use of register data we could obtain information with complete coverage during the entire follow-up period. This study was able to reproduce findings from other studies on a population level using a well-established and rich twin cohort. It has been claimed that twins are dissimilar from the general population, but previous work has shown that twins are not significantly different from singletons (Evans and Martin, 2000). A large Swedish study that compared twins and singletons found that there were only small differences in cognition and attained education between the two groups and further showed no difference in vocational career (Hjern et al., 2012).

This study also has several limitations. We used only educational differences as an indicator of SES, which may not be directly comparable to socioeconomic measures used in other studies. Our findings are in line with two previous studies in Swedish twins where unemployment or lengthy working hours was associated with increased mortality risk for both men and women (Nylén et al., 2001; Voss et al., 2004), indicating that the increased risk of mortality holds for several measures of SES. Using socioeconomic variables over time could entail challenges as educational opportunities differ over time. The birth cohort in this study was a part of the educational reforms that were implemented in the 50’s and 60’s (Larsson and Westberg, 2015), but the associations were robust when stratifying on birth cohort. Thus, by using a study population with a wide birth year range, we could show that the association between education and mortality is, in fact, stable before and after the educational reforms. Another limitation of this study is the measurement of geographical data, namely that we only have geographical information from birth and until 1973, thus not covering the entire adult lifespan from 1974 onwards. In addition, we used moving across county lines as our geographical measurement which is a rather crude measurement, and thus did not consider how far they moved. The information on geographical movement was self-reported places lived, from birth to 1973, which means we also catch those moving out of and then back to their birth county, amplifying the chance of being registered as being geographically mobile regardless of specific patterns.

Because of the birth years of participants in this study, there is a potential that the findings might not be generalizable to cohorts growing up today. The participants in this cohort grew up in a society where socioeconomic class differences were widespread. However, even with the current social safety net in Sweden, wide differences is socioeconomic status remain, as does the SES-health gradient (Gastwirth, 2014).

## 7. Conclusions

Through using rich longitudinal data, we contribute further insights regarding the long-term effects of education and geographic mobility on health. Both genetic propensity for education and attained education predicted geographic mobility, with higher education indicating a higher mobility. Furthermore, our results show that attained education explains the relationship between geographic mobility and mortality. Importantly, even if genetic propensity for education was associated with both attained education and geographic mobility, it was only attained education that was associated with a lower risk of mortality. This result indicates that higher education is more important than one’s genetic predisposition for educational achievement, highlighting the importance and potential preventive value of working towards availability of better education for all.

## Supplementary Material

Appendix A. Supplementary data

## Figures and Tables

**Table 1:** Descriptive statistics of the study population.

	Men N = 6694 (47.10%)		Women N = 7517 (52.90%)	
	Not mobile N = 4603 (68.76%)	Mobile N = 2091 (31.24%)	Not mobile N = 4712 (62.68%)	Mobile N = 2805 (37.32%)

**Birth cohort N (%)**				
**1**(1926–1940)	1288 (27.98%)	1058 (50.60%)	1322 (28.06%)	1205 (42.96%)
**2**(1941–1955)	3315 (72.02%)	1033 (49.40%)	3390 (71.94%)	1600 (57.04%)
**Education (ISCED) N (%)**				
Primary	1229 (27.50%)	378 (18.51%)	1144 (24.87%)	558 (20.31%)
Lower secondary	1431 (32.02%)	420 (20.57%)	1504 (32.70%)	743 (27.05%)
Upper secondary or post-secondary non-tertiary	979 (21.91%)	408 (19.98%)	894 (19.43%)	384 (13.98%)
Short-cycle tertiary	287 (6.42%)	200 (9.79%)	413 (8.98%)	320 (11.65%)
University degree (bachelor’s or above)	543 (12.15%)	636 (31.15%)	645 (14.02%)	742 (27.01%)
**PGS for education M(SD)**	0.697 (0.27)	0.785 (0.26)	0.691 (0.27)	0.741 (0.27)
**Vital status N (%)**				
Alive	3291 (71.50%)	1297 (62.03%)	3690 (78.31%)	2026 (72.23%)
Dead	1312 (28.50%)	794 (37.97%)	1022 (21.69%)	779 (27.77%)

Descriptive statistics for all individuals by sex and geographic mobility. Statistics are presented as number (%) of individuals for categorical variables and mean level (SD) for continuous variables. N = 14,211. Education (n = 13,858). PGS_Edu_ (n = 8094). *N* number, *ISCED* International Standard Classification of Education, *SD* standard deviation, *PGS* polygenic score.

**Table 2:** Odds of geographic mobility as a function of attained educational level and PGS_Edu_.

Total sample	Unadjusted model		Adjusted model^a^	
	Odds Ratio	[CI 95%]	Odds Ratio	[CI 95%]

Independent effect model				
Education	1.32	**(1.29–1.36)**	**1.59**	**(1.54–1.64)**
PGS_Edu_	1.28	**(1.21–1.35)**	**1.29**	**(1.23–1.37)**
Joint effect model				
Education			**1.57**	**(1.51–1.64)**
PGS_Edu_			**1.12**	**(1.06–1.19)**
				
**Men born 1926–1940**				
Independent effect model				
Education	**1.74**	**(1.62–1.88)**	**1.75**	**(1.63–1.88)**
PGS_Edu_	**1.33**	**(1.18–1.51)**	**1.33**	**(1.18–1.52)**
Joint effect model	1			
Education			**1.70**	**(1.53–1.88)**
PGS_Edu_			1.13	(0.99–1.29)
**Women born 1926–1940**				
Independent effect model				
Education	**1.62**	**(1.51–1.73)**	**1.63**	**(1.52–1.74)**
PGS_Edu_	**1.17**	**(1.03–1.33)**	**1.18**	**(1.04–1.34)**
Joint effect model				
Education			**1.70**	**(1.53–1.88)**
PGS_Edu_			1.05	(0.91–1.20)
**Men born 1941–1955**				
Independent effect model				
Education	**1.53**	**(1.44–1.63)**	**1.72**	**(1.61–1.83)**
PGS_Edu_	**1.43**	**(1.29–1.58)**	**1.50**	**(1.35–1.66)**
Joint effect model				
Education			**1.63**	**(1.50–1.77)**
PGS_Edu_			**1.31**	**(1.17–1.47)**
**Women born 1941–1955**				
Independent effect model				
Education	**1.26**	**(1.20–1.33)**	**1.49**	**(1.41–1.57)**
PGS_Edu_	**1.23**	**(1.13–1.34)**	**1.27**	**(1.16–1.39)**
Joint effect model				
Education			**1.50**	**(1.40–1.61)**
PGS_Edu_			1.09	(0.99–1.20)

Education (n = 13,858). PGS_Edu_ (n = 8094). Joint model (n = 7741).

a Adjusted model is adjusted for both sex and birth year. Odds ratios (95% confidence intervals) of geographic mobility as a function of attained educational level and PGS_Edu_ stratified by total sample, cohort and sex. Statistically significant estimates are presented in bold. Independent model contains either Education or PGS_Edu_ as predictor of geographic mobility. Joint effect models contains both Education and PGS_Edu_ as predictors of geographic mobility. *PGS_Edu_* Polygenic score for education.

## Data Availability

The data can be applied for from the Swedish Twin Registry: https://ki.se/en/research/swedish-twin-registry-for-researchers

## Abbreviations:

PGS_Edu_: Polygenic score for education
SES: Socioeconomic status.

## References

[R1] Abdellaoui A, Hugh-Jones D, Yengo L, Kemper KE, Nivard MG, Veul L, , 2019. Genetic correlates of social stratification in Great Britain. Nat. Human Behav. 3, 1332–1342. 31636407 10.1038/s41562-019-0757-5

[R2] Befolkningsstatistik, 2022. Statistics Sweden.

[R3] Belsky DW, Caspi A, Arseneault L, Corcoran DL, Domingue BW, Harris KM, , 2019. Genetics and the geography of health, behaviour and attainment. Nat. Human Behav. 3, 576–586. 30962612 10.1038/s41562-019-0562-1 PMC6565482

[R4] Belsky DW, Moffitt TE, Corcoran DL, Domingue B, Harrington HL, Hogan S, , 2016. The genetics of success: how single-nucleotide polymorphisms associated with educational attainment relate to life-course development. Psychol. Sci. 27, 957–972. 27251486 10.1177/0956797616643070 PMC4946990

[R5] Branigan AR, McCallum KJ, Freese J, 2013. Variation in the heritability of educational attainment: an international meta-analysis. Soc. Forces 92, 109–140.

[R6] Breen R, 2004. Social Mobility in Europe. Oxford University Press.

[R7] Chen Y, Rosenthal SS, 2008. Local amenities and life-cycle migration: do people move for jobs or fun? J. Urban Econ. 64, 519–537.

[R8] Colodro-Conde L, Rijsdijk F, Tornero-Gómez MJ, Sánchez-Romera JF, Ordoñana JR, 2015. Equality in educational policy and the heritability of educational attainment. PLoS One 10. 10.1371/journal.pone.0143796 PMC4664401 26618539

[R9] Curtis S, Setia MS, Quesnel-Vallee A, 2009. Socio-geographic mobility and health status: a longitudinal analysis using the National Population Health Survey of Canada. Soc. Sci. Med. 69, 1845–1853. 19822386 10.1016/j.socscimed.2009.08.004 PMC3762746

[R10] Deary IJ, Strand S, Smith P, Fernandes C, 2007. Intelligence and educational achievement. Intelligence 35, 13–21.

[R11] Ericsson M, 2019. Socioeconomic Influences on Late-Life Health and Mortality: Exploring Genetic and Environmental Interplay.

[R12] Ericsson M, Pedersen NL, Johansson ALV, Fors S, Dahl Aslan AK, 2019. Life-course socioeconomic differences and social mobility in preventable and non-preventable mortality: a study of Swedish twins. Int. J. Epidemiol. 48, 1701–1709. 30929008 10.1093/ije/dyz042 PMC6857748

[R13] Eurostat O, 2015. ISCED 2011 Operational Manual: Guidelines for Classifying National Education Programmes and Related Qualifications.

[R14] Evans DM, Martin NG, 2000. Are Twins the Same as Singletons? the Validity of Twin Studies

[R15] Fors S, Wastesson JW, Morin L, 2021. Growing income-based inequalities in old-age life expectancy in Sweden, 2006–2015. Demography 58, 2117–2138. 34528078 10.1215/00703370-9456514

[R16] Foverskov E, Holm A, 2016. Socioeconomic inequality in health in the British household panel: tests of the social causation, health selection and the indirect selection hypothesis using dynamic fixed effects panel models. Soc. Sci. Med. 150, 172–183. 26761376 10.1016/j.socscimed.2015.12.021

[R17] Gastwirth JL, 2014. Median-based measures of inequality: reassessing the increase in income inequality in the U.S. and Sweden. Stat. J. IAOS 30, 311–320.

[R18] Haley A, 2017. Defining geographical mobility: perspectives from higher education. Geoforum 83, 50–59.

[R19] Hjern A, Ekeus C, Rasmussen F, Lindblad F, 2012. Educational achievement and vocational career in twins - a Swedish national cohort study. Acta Paediatr., Int. J. Paediatr. 101, 591–596. 10.1111/j.1651-2227.2012.02636.x 22353254

[R20] Hoffmann R, Kröger H, Geyer S, 2019. Social causation versus health selection in the life course: does their relative importance differ by dimension of SES? Soc. Indicat. Res. 141, 1341–1367.

[R21] Huisman M, Kunst AE, Andersen O, Bopp M, Borgan JK, Borrell C, , 2004. Socioeconomic inequalities in mortality among elderly people in 11 European populations. J. Epidemiol. Commun. Health 58, 468–475. 10.1136/jech.2003.010496 PMC1732782 15143114

[R22] Härkönen J, Bihagen E, 2011. Occupational attainment and career progression in Sweden. Eur. Soc. 13, 451–479.

[R23] Larsson E, Westberg J, 2015. Utbildningshistoria : en introduktion. Studentlitteratur.

[R24] Lee JJ, Wedow R, Okbay A, Kong E, Maghzian O, Zacher M, , 2018. Gene discovery and polygenic prediction from a genome-wide association study of educational attainment in 1.1 million individuals. Nat. Genet. 50, 1112–1121. 30038396 10.1038/s41588-018-0147-3 PMC6393768

[R25] Lichtenstein P, De Faire U, Floderus B, Svartengren M, Svedberg P, Pedersen NL, 2002. The Swedish Twin Registry: a unique resource for clinical, epidemiological and genetic studies. J. Intern. Med. 184–205. 12270000 10.1046/j.1365-2796.2002.01032.x

[R26] Lichtenstein P, Sullivan PF, Cnattingius S, Gatz M, Johansson S, Carlström E, , 2006. The Swedish twin registry in the third millennium: an update. Twin Res. Hum. Genet. 9, 875–882. 10.1375/183242706779462444 17254424

[R27] Mackenbach JP, Kulhánová I, Bopp M, Deboosere P, Eikemo TA, Hoffmann R, , 2015. Variations in the relation between education and cause-specific mortality in 19 European populations: a test of the “fundamental causes” theory of social inequalities in health. Soc. Sci. Med. 127, 51–62. 24932917 10.1016/j.socscimed.2014.05.021

[R28] Mackenbach JP, Looman CWN, Artnik B, Bopp M, Deboosere P, Dibben C, , 2017. ‘Fundamental causes’ of inequalities in mortality: an empirical test of the theory in 20 European populations. Sociol. Health Illness 39, 1117–1133. 10.1111/1467-9566.12562 28369947

[R29] Mackenbach JP, Stirbu I, Roskam A-JR, Schaap MM, Menvielle G, Leinsalu M, , 2008. Socioeconomic inequalities in health in 22 European countries. N. Engl. J. Med. 358, 2468–2481. 18525043 10.1056/NEJMsa0707519

[R30] Magnusson PK, Almqvist C, Rahman I, Ganna A, Viktorin A, Walum H, , 2013. The Swedish Twin Registry: establishment of a biobank and other recent developments. Twin Res. Hum. Genet. 16, 317–329. 10.1017/thg.2012.104 23137839

[R31] Mirowsky J, Ross CE, Mirowsky J, Ross CE, 2005. Send correspondence to. Ageing Int. 27–62.

[R32] Mishra GD, Kuh D, Ben-Shlomo Y, 2015. International Encyclopedia of the Social & Behavioral Sciences. In: Life Course Epidemiology, second ed. Elsevier Inc, pp. 67–75.

[R33] Nylén L, Voss M, Floderus B, 2001. Mortality among women and men relative to unemployment, part time work, overtime work, and extra work: a study based on data from the Swedish twin registry. Occup. Environ. Med. 58, 52–57. 11119635 10.1136/oem.58.1.52 PMC1740025

[R34] Okbay A, 2017. Essays on Genetics and the Social Sciences. Erasmus Universiteit Rotterdam (EUR).

[R35] Okbay A, Wu Y, Wang N, Jayashankar H, Bennett M, Nehzati SM, , 2022. Polygenic prediction of educational attainment within and between families from genome-wide association analyses in 3 million individuals. Nat. Genet. 54, 437–449. 35361970 10.1038/s41588-022-01016-z PMC9005349

[R36] Organization WH, 2021. World Report on Ageing and Health. World Health Organization.

[R37] Pedersen NL, Lichtenstein P, Svedberg P, 2002. The Swedish twin registry in the third millennium. Twin Res. 5, 427–432. 12537870 10.1375/136905202320906219

[R38] Phelan JC, Link BG, Diez-Roux A, Levin B, Phelan J, 2004. Fundamental causes” of social inequalities in mortality: a test of the theory*. J. Health Soc. Behav..Sep 45 (3), 265–285. 15595507 10.1177/002214650404500303

[R39] Plomin R, Deary IJ, 2015. Genetics and intelligence differences: five special findings. Mol. Psychiatr. 98–108 (Nature Publishing Group). 10.1038/mp.2014.105 PMC4270739 25224258

[R40] Pärna K, Nolte IM, Snieder H, Fischer K, E.B.R.T., Marnetto D, , 2022. A principal component informed approach to address polygenic risk score transferability across European cohorts. Front. Genet. 13. 10.3389/fgene.2022.899523 PMC9340200 35923706

[R41] Smith JP, 2007. The impact of socioeconomic status on health over the life-course. Source: J. Hum. Resour. 739–764.

[R42] Socialstyrelsen, 2020. In: Wigzell O (Ed.), Vård Och Omsorg Om Äldre Socialstyrelsen. Socialstyrelsen, pp. 3–121 (2020).

[R43] StataCorp, L.J.C.S., 2015. Stata Statistical Software (Version Release 14). Author, College Station, TX, 464, 465. 14.

[R44] Voss M, Nylén L, Floderus B, Diderichsen F, Terry PD, 2004. Unemployment and early cause-specific mortality: a study based on the Swedish twin registry. Am. J. Publ. Health 94, 2155–2161. 10.2105/ajph.94.12.2155 PMC1448606 15569968

[R45] Zagai U, Lichtenstein P, Pedersen NL, Magnusson PKE, 2019. The Swedish twin registry: content and management as a research infrastructure. Twin Res. Hum. Genet. 22, 672–680. 31747977 10.1017/thg.2019.99

