# Supplementary material for: Influences of genetically predicted and attained education on geographic mobility and their association with mortality
Source: Soc Sci Med. Author manuscript; Available in PMC 2024 Oct 31. (PMC11526346; doi:10.1016/j.socscimed.2023.115882)
Supplement: Appendix A. Supplementary data [file NIHMS2021405-supplement-Appendix_A__Supplementary_data.docx]

**Supplementary section**

*PGS for education* was measured by DNA collection from the Karolinska Institutet (KI) biobank (Magnusson et al., 2013). The study population was restricted to twins that participated in SALT. The TwinGene project was aimed at SALT participants with a peak in participation for individuals born 1936-1940. This project is a resource equipped to explore the molecular influences on a wide-ranging spectrum of health-related diseases and traits, as well as gene-environment interactions. The DNA collection consisted of blood samples where clinical blood chemistry assessments were performed by the Karolinska University Laboratory (Magnusson et al., 2013). The Screening Across the Lifespan Twin Study: the Younger (SALT-Y) is a project where the population was the younger part of the SALT cohort born between 1943-1958. These twins belong to the active workforce, and it was estimated that an important but probable factor for the low participation in the TwinGene project was limited time for visits to a healthcare facility throughout weekdays to donate blood. The SALT-Y project collected DNA by saliva DNA kits, specifically, the Oragene DNA OG-500 kits (Magnusson et al., 2013).

The polygenic scores are calculated according to individual level genotype profile (target sample) and summary statistics from a relevant Genome Wide Association study (GWAS; discovery sample). GWAS are a research approach used to identify genomic variants across the genome associated with a risk of a disease or a trait. We have used the “EA3 GWAS” by Lee and colleagues, including 1.1 million individuals (Lee et al., 2018). All genetic variants included in the HapMap3 reference, present in at least 1% of the target data, and imputed with good quality (INFO score >0.8) were included for analyses. To deal with linkage disequilibrium, we applied SBayesR Bayesian shrinkage to the discovery data (Lloyd-Jones et al., 2019). A polygenic scores was then computed in Plink2, by summing the number of effect alleles (0, 1 or 2) at each position in the genome, weighted by their effect in the GWAS of the trait.

To control for genetic ancestry, principal components were computed on pooled data from TwinGene and SALT-Y (using genetic variants present in at least 5% of the sample, and with INFO score >0.95) and included in the analyses.

**Table S1.**The association between the PGS_Edu_ and attained education, stratified by geographic mobility

| **Education** | **Not mobile** | | **Mobile** | |
| --- | --- | --- | --- | --- |
|  | β | [95% CI] | β | [95% CI] |
| **Born 1926-1940** |  |  |  |  |
| **Men** |  |  |  |  |
| PGS_Edu_ | **0.15** | **0.06-0.24** | **0.48** | **0.36-0.60** |
| **Women** |  |  |  |  |
| PGS_Edu_ | **0.14** | **0.05-0.23** | **0.38** | **0.27-0.50** |
| **Born 1941-1955** |  |  |  |  |
| **Men** |  |  |  |  |
| PGS_Edu_ | **0.26** | **0.19-0.32** | **0.39** | **0.29-0.49** |
| **Women** |  |  |  |  |
| PGS_Edu_ | **0.30** | **0.25-0.36** | **0.44** | **0.36-0.52** |

Linear regression investigating the effect PGS_Edu_ has on attained education depending on geographic mobility (not mobile/mobile) stratified on gender and cohorts. Statistically significant estimates are presented in bold. *PGS_Edu_* polygenic score for education.

**Table S2.** Risk of mortality in relation to geographic mobility, attained education and PGS_Edu_ stratified on cohort and sex

| **Risk of mortality** | **(HR)** | **[CI 95%]** | **P-value** |
| --- | --- | --- | --- |
| **Men born 1926-1940** | |  |  |
| *Independent models* |  |  |  |
| Geographic mobility | 0.97 | (0.87-1.07) | 0.525 |
| Attained education | **0.94** | **(0.90-0.98)** | 0.001 |
| PGS_Edu_ | 0.99 | (0.92-1.07) | 0.804 |
| *Joint effect models* |  |  |  |
| Geographic mobility | 1.00 | (0.85-1.17) | 0.959 |
| Attained education | 0.95 | (0.90-1.01) | 0.100 |
| PGS_Edu_ | 1.01 | (0.93-1.09) | 0.774 |
| **Women born 1926-1940** | |  |  |
| *Independent models* |  |  |  |
| Geographic mobility | 0.94 | (0.84-1.06) | 0.310 |
| Attained education | **0.94** | **(0.90-0.99)** | 0.014 |
| PGS_Edu_ | 1.02 | (0.93-1.12) | 0.730 |
| *Joint effect models* |  |  |  |
| Geographic mobility | 1.03 | (0.85-1.23) | 0.800 |
| Attained education | **0.91** | **(0.85-0.99)** | 0.020 |
| PGS_Edu_ | 1.05 | (0.95-1.16) | 0.335 |
| **Men born 1941-1955** |  |  |  |
| *Independent models* |  |  |  |
| Geographic mobility | **0.82** | **(0.69-0.98)** | 0.025 |
| Attained education | **0.87** | **(0.82-0.92)** | 0.000 |
| PGS_Edu_ | 0.90 | (0.80-1.01) | 0.085 |
| *Joint effect models* |  |  |  |
| Geographic mobility | 0.82 | (0.61-1.05) | 0.103 |
| Attained education | **0.90** | **(0.82-0.99)** | 0.024 |
| PGS_Edu_ | 0.96 | (0.84-1.09) | 0.533 |
| **Women born 1941-1955** | |  |  |
| *Independent models* |  |  |  |
| Geographic mobility | 1.00 | (0.84-1.18) | 0.984 |
| Attained education | **0.86** | **(0.81-0.92)** | 0.000 |
| PGS_Edu_ | 0.91 | (0.81-1.02) | 0.112 |
| *Joint effect models* |  |  |  |
| Geographic mobility | **1.44** | **(1.11-1.86)** | 0.006 |
| Attained education | **0.84** | **(0.77-0.93)** | 0.000 |
| PGS_Edu_ | 0.93 | (0.82-1.05) | 0.252 |

Cox regression investigating the risk, hazard rate ratios and 95% confidence intervals of mortality as a function of geographic mobility, attained education and PGS_Edu_ stratified on subsamples by cohort and sex. All models are adjusted for birth year. Independent model contains either geographic mobility, attained education or PGS_Edu_ as risk of mortality. Joint effect models contains geographic mobility, attained education and PGS_Edu_ together as risk of mortality. Statistically significant estimates are presented in bold. *PGS_Edu_* polygenic score for education.

**Table S3.** Cox regression investigating risk of mortality as an effect on geographic mobility stratified by education

| Educational level | | Geographically mobile | |
| --- | --- | --- | --- |
| **Risk of mortality** | **(HR)** | **[CI 95%]** | **P-value** |
| **Cohort 1 Men** |  |  |  |
| Primary | 0.90 | (0.69-1.18) | 0.445 |
| Lower secondary | 1.13 | (0.84-1.52) | 0.420 |
| Upper secondary | 1.11 | (0.74-1.68) | 0.612 |
| Short-cycle tertiary | 1.08 | (0.59.1.95) | 0.811 |
| University | 0.83 | (0.50-1.39) | 0.480 |
| **Cohort 1 Women** |  |  |  |
| Primary | 0.98 | (0.72-1.32) | 0.875 |
| Lower secondary | 0.87 | (0.65-1.17) | 0.355 |
| Upper secondary | 1.63 | (0.77-3.45) | 0.201 |
| Short-cycle tertiary | 0.89 | (0.39-2.02) | 0.782 |
| University | 2.62 | (0.82-8.46) | 0.105 |
| **Cohort 2 Men** |  |  |  |
| Primary | 0.92 | (0.48-1.74) | 0.793 |
| Lower secondary | 0.72 | (0.43-1.19) | 0.197 |
| Upper secondary | 0.65 | (0.34-1.23) | 1.184 |
| Short-cycle tertiary | 0.74 | (0.31-1.74) | 0.489 |
| University | 1.00 | (0.59-1.71) | 0.993 |
| **Cohort 2 Women** |  |  |  |
| Primary | **2.42** | **(1.44-4.19)** | 0.001 |
| Lower secondary | **1.95** | **(1.25-3.06)** | 0.003 |
| Upper secondary | 0.94 | (0.54-1.64) | 0.840 |
| Short-cycle tertiary | 0.94 | (0.40-1.78) | 0.652 |
| University | 0.93 | (0.52-1.67) | 0.811 |

Cox regression investigating the risk, hazard rate ratios and 95% confidence intervals of mortality as a function of geographic mobility stratified on attained education for subsamples by cohort and sex. All models are adjusted for birth year. Statistically significant estimates are presented in bold. *PGS_Edu_* polygenic score for education.

**Table S4.** Risk of mortality in relation to geographic mobility, attained education and PGS_Edu_ for those who moved after age 18 and were at least 25 years old in 1973

| **Risk of mortality** | **Hazard Ratio (HR)** | **[CI 95%]** | **P-value** |
| --- | --- | --- | --- |
|  |  |  |  |
| *Independent models* |  |  |  |
| Geographic mobility | **0.93** | **(0.87-0.99)** | **0.036** |
| Attained education | **0.92** | **(0.90-0.95)** | **0.000** |
| PGS_Edu_ | 0.98 | (0.93-1.03) | 0.459 |
| *Joint effect models* |  |  |  |
| Geographic mobility | 1.02 | (0.92-1.14) | 0.706 |
| Attained education | **0.93** | **(0.88-0.96)** | **0.000** |
| PGS_Edu_ | 1.00 | (0.95-1.06) | 0.763 |

Cox regression investigating the risk, hazard rate ratios and 95% confidence intervals of mortality as a function of geographic mobility, attained education and PGS_Edu_ for those who moved after the age of 18 and who were at least 25 years old in 1973. All models are adjusted for sex and birth year. Independent model contains either geographic mobility, attained education or PGS_Edu_ as risk of mortality. Joint effect models contains geographic mobility, attained education and PGS_Edu_ together as risk of mortality. Statistically significant estimates are presented in bold. *PGS_Edu_* polygenic score for education.

**Table S5.** Risk of mortality in relation to geographic mobility, attained education and PGS_Edu_ stratified on cohort and sex

| **Risk of mortality** | **Hazard Ratio (HR)** | **[CI 95%]** | **P-value** |
| --- | --- | --- | --- |
| **Men born 1926-1940** |  |  |  |
| *Independent models* |  |  |  |
| Geographic mobility | 0.96 | (0.87-1.07) | 0.481 |
| Attained education | **0.93** | **(0.90-0.98)** | **0.001** |
| PGS_Edu_ | 0.99 | (0.92-1.07) | 0.856 |
| *Joint effect models* |  |  |  |
| Geographic mobility | 1.00 | (0.85-1.18) | 0.968 |
| Attained education | 0.95 | (0.89-1.01) | 0.089 |
| PGS_Edu_ | 1.01 | (0.94-1.10) | 0.718 |
| **Women born 1926-1940** |  |  |  |
| *Independent models* |  |  |  |
| Geographic mobility | 0.93 | (0.83-1.04) | 0.201 |
| Attained education | **0.95** | **(0.90-0.99)** | **0.016** |
| PGS_Edu_ | 1.01 | (0.92-1.12) | 0.786 |
| *Joint effect models* |  |  |  |
| Geographic mobility | 1.01 | (0.84-1.22) | 0.890 |
| Attained education | 0.92 | **(0.85-0.99)** | **0.022** |
| PGS_Edu_ | 1.05 | (0.95-1.16) | 0.347 |
| **Men born 1941-1955** |  |  |  |
| *Independent models* |  |  |  |
| Geographic mobility | **0.81** | **(0.67-0.98)** | **0.032** |
| Attained education | **0.89** | **(0.84-0.96)** | **0.001** |
| PGS_Edu_ | 0.94 | (0.82-1.08) | 0.371 |
| *Joint effect models* |  |  |  |
| Geographic mobility | 0.84 | (0.62-1.13) | 0.249 |
| Attained education | 0.96 | (0.87-1.06) | 0.440 |
| PGS_Edu_ | 0.97 | (0.83-1.14) | 0.700 |
| **Women born 1941-1955** |  |  |  |
| *Independent models* |  |  |  |
| Geographic mobility | 0.97 | (0.80-1.17) | 0.767 |
| Attained education | **0.86** | **(0.80-0.92)** | **0.000** |
| PGS_Edu_ | 0.92 | (0.80-1.06) | 0.233 |
| *Joint effect models* |  |  |  |
| Geographic mobility | 1.41 | (1.04-1.91) | 0.023 |
| Attained education | **0.83** | **(0.74-0.92)** | **0.000** |
| PGS_Edu_ | 0.95 | (0.82-1.09) | 0.453 |

Cox regression investigating the risk, hazard rate ratios and 95% confidence intervals of mortality as a function of geographic mobility, attained education and PGS_Edu_ stratified on subsamples by cohort and sex for those who moved after the age of 25. All models are adjusted for birth year. Independent model contains either geographic mobility, attained education or PGS_Edu_ as risk of mortality. Joint effect models contains geographic mobility, attained education and PGS_Edu_ together as risk of mortality. Statistically significant estimates are presented in bold. *PGS_Edu_* polygenic score for education.

**Table S6.** Odds of geographic mobility as a function of attained educational level and PGS_Edu_ adjusted for parental SES.

|  | **Unadjusted model** | | **Adjusted model*** | |
| --- | --- | --- | --- | --- |
| **Total sample** | **Odds Ratio** | **[CI 95%]** | **Odds Ratio** | **[CI 95%]** |
| *Independent effect model* |  |  |  |  |
| Education | **1.32** | **(1.29–1.36)** | **1.53** | **(1.47–1.59)** |
| PGS_Edu_ | **1.28** | **(1.21–1.35)** | **1.27** | **(1.18–1.37)** |
| *Joint effect model* |  |  |  |  |
| Education |  |  | **1.52** | **(1.44–1.62)** |
| PGS_Edu_ |  |  | **1.14** | **(1.05–1.23)** |

Education (n=8935). PGS_Edu_ (n=8094). Joint effect model (n=4587).

*Adjusted model is adjusted for both sex, birth year and parental SES. Odds ratios (95% confidence intervals) of geographic mobility as a function of attained educational level and PGS_Edu_ stratified by total sample, cohort and sex. Statistically significant estimates are presented in bold. Independent model contains either Education or PGS_Edu_ as predictor of geographic mobility. Joint effect models contains both Education and PGS_Edu_ as predictors of geographic mobility. *PGS_Edu_* Polygenic score for education, *SES* socioeconomic status.

**Table S7.** Risk of mortality in relation to geographic mobility, attained education and PGS_Edu_ adjusted for parental SES

| **Risk of mortality** |  | **HR** | **[CI 95%]** | **P-value** |
| --- | --- | --- | --- | --- |
| *Independent models* | |  |  |  |
| **Geographic mobility** |  | 0.95 | (0.86–1.03) | 0.242 |
| **Attained education** | | **0.91** | **(0.89–0.94)** | **0.000** |
| **PGS_Edu_** |  | 0.98 | (0.91–1.05) | 0.584 |
| *Joint effect models* | |  |  |  |
| **Geographic mobility** |  | 1.06 | (0.91–1.24) | 0.445 |
| **Attained education** | | **0.88** | **(0.83–0.93)** | **0.000** |
| **PGS_Edu_** |  | 1.01 | (0.94–1.09) | 0.705 |

Education (n=8935). PGS_Edu_ (n=8094). Joint effect model (n=4587).

Cox regression investigating the risk, hazard rate ratios and 95% confidence intervals of mortality as a function of geographic mobility, attained education and PGS_Edu_. All models are adjusted for sex, birth year and parental SES. Statistically significant estimates are presented in bold. *PGS_Edu_* Polygenic score for education, *SES* socioeconomic status.

**Table S8.** Odds of geographic mobility as a function of attained educational level and PGS_Edu_, using the sample with both attained education and PGS_Edu_ (N=7741), same model as Table 2

|  | **Independent model** | | | **Adjusted model*** | | |
| --- | --- | --- | --- | --- | --- | --- |
|  | **Odds Ratio** | **P-value** | **[CI 95%]** | **Odds Ratio** | **P-value** | **[CI 95%]** |
| Independent model |  |  |  |  |  |  |
| **Education** | **1.33** | **0.000** | **1.28-1.38** | **1.60** | **0.000** | **1.53-1.66** |
| **PGS_Edu_** | **1.28** | **0.000** | **1.21-1.34** | **1.30** | **0.000** | **1.23-1.37** |

*Adjusted model is adjusted for both sex and birth year. Odds ratios (95% confidence intervals) of geographic mobility as a function of attained educational level and PGS_Edu_. Statistically significant estimates are presented in bold. Independent model contains either Education or PGS_Edu_ as predictor of geographic mobility. *PGS_Edu_* Polygenic score for education.

**Table S9.** Risk of mortality in relation to geographic mobility, attained education or PGS_Edu_ using the sample with both attained education and PGS_Edu_ (N=7741), same model as Figure 1

| **Risk of mortality** | **Hazard Ratio (HR)** | **P-value** | **[CI 95%]** |
| --- | --- | --- | --- |
| *Independent model* |  |  |  |
| **Geographic mobility** | 0.94 | 0.157 | (0.85-1.03) |
| **Attained education** | **0.91** | **0.000** | **(0.88-0.95)** |
| **PGS_Edu_** | 0.97 | 0.211 | (0.92-1.02) |

Cox regression investigating the risk. Hazard rate ratios and 95% confidence intervals of mortality as a function of geographic mobility, attained education or PGS_Edu_. All models include individuals with complete data on geographic mobility, attained education and PGS_Edu_. All models are adjusted for sex and birth year. Statistically significant estimates are presented in bold. *PGS_Edu_* Polygenic score for education. *SES* socioeconomic status.

References:

Lee, J.J., Wedow, R., Okbay, A., Kong, E., Maghzian, O., Zacher, M., et al. (2018). Gene discovery and polygenic prediction from a genome-wide association study of educational attainment in 1.1 million individuals. *Nature Genetics,* 50, 1112-1121.

Lloyd-Jones, L.R., Zeng, J., Sidorenko, J., Yengo, L., Moser, G., Kemper, K.E., et al. (2019). Improved polygenic prediction by Bayesian multiple regression on summary statistics. *Nat Commun,* 10, 5086.

Magnusson, P.K.E., Almqvist, C., Rahman, I., Ganna, A., Viktorin, A., Walum, H., et al. (2013). The swedish twin registry: Establishment of a biobank and other recent developments. *Twin Research and Human Genetics,* 16, 317-329.
